# Oxidative stress and visual system: a review

**DOI:** 10.17179/excli2022-4663

**Published:** 2022-03-01

**Authors:** Samanta Taurone, Massimo Ralli, Marco Artico, Valentina Noemi Madia, Susanna Scarpa, Stefania Annarita Nottola, Antonio Maconi, Marta Betti, Pietro Familiari, Marcella Nebbioso, Roberta Costi, Alessandra Micera

**Affiliations:** 1 IRCCS - Fondazione Bietti, Rome, Italy; 2 Department of Sensory Organs, "Sapienza" University of Rome, Rome, Italy; 3 Department of Drug Chemistry and Technology, "Sapienza" University of Rome, Rome, Italy; 4 Department of Experimental Medicine, "Sapienza" University of Rome, Italy; 5 Department of Anatomy, Histology, Forensic Medicine and Orthopaedics, "Sapienza" University of Rome, Rome, Italy; 6 Research Training Innovation Infrastructure, Research and Innovation Department, Azienda Ospedaliera SS. Antonio e Biagio e Cesare Arrigo, Alessandria, Italy; 7 Department of Human Neurosciences, "Sapienza" University of Rome, Rome, Italy

**Keywords:** alpha-lipoic acid (ALA), antioxidants, retina and optic nerve aging, superoxide dismutase (SOD)

## Abstract

Different types of tissues respond differently to the action of oxidative stress. The visual system is very sensitive to oxidative action due to continuous exposure to light. In consideration of the growing interest of scientific studies towards various compounds endowed with antioxidant and anti-inflammatory properties, we performed a review of the literature focusing on the use of some antioxidant molecules for the treatment of conditions affecting the visual system. In this study, we focused on the ability of two antioxidant agents, the small molecule α-lipoic acid (ALA) and the enzyme superoxide dismutase (SOD), to influence the neurodegenerative physiological processes related to aging and oxidative stress affecting the ocular segment. The literature data report that ALA and SOD can protect against neurodegenerative effects both the optic nerve and retina and, if administered together, they are able to lower the levels of oxidative stress, thus preventing neurodegeneration and reducing the apoptotic process.

## Oxidative Stress

The term oxidative stress refers to the set of alterations that occurs after the exposure of biological tissues, cells, and macromolecules, to an excess of oxidizing agents (Kudryavtseva et al., 2016[30]; Sies, 1993[49]; Halliwell and Gutteridge, 2007[21]). Reactive radical species are physiologically produced by the cell during metabolism, however, their excessive concentration due to an over-production or as a consequence of decreased levels of antioxidants can be harmful to cells (Halliwell and Gutteridge, 2007[21]; Li et al., 2016[32]). The substances responsible for oxidative stress are not only free radicals, but also other elements that have a different chemical structure but are capable to exert an oxidative action on cells. These are the so-called "non-radical oxidizing chemical species" which, unlike free radicals, that possess an unpaired electron at the orbital level, have their electrons mostly distributed in pairs and engaged in covalent bonds. An example is represented by the hydrogen peroxide (H_2_O_2_) (Li et al., 2016[32]; Sies, 2015[48]). Despite being structurally different, free radicals and non-radical chemical species share an intrinsic characteristic which is their oxidizing capacity. Frequently, when addressing the subject of free radicals reference is made to molecules which contain oxygen such as the radical anion superoxide (O_2_^-^), hydrogen peroxide (H_2_O_2_), and hydroxyl radicals (Li et al., 2016[32]; Sies, 2015[48]; Davies, 1995[14]). However, the oxidizing action may be exerted even by free radicals containing carbon or nitrogen (the so-called oxygen-free radicals) (Sies, 2015[48]).

### Reactive species (RS)

Based on their chemical nature, reactive species (RS) are divided into reactive oxygen species (ROS), reactive nitrogen species (RNS), reactive chlorine species (RClS), reactive sulfur species (RSS), and reactive bromine species (RBrS). The most important ones, in both quantitative and qualitative terms, are ROS and RNS. In all aerobic organisms there is a delicate redox balance between the oxidizing substances produced, including ROS, and the antioxidant defence system which has the function of preventing and repairing the damage induced by them (Sies, 2015[48]; Davies, 1995[14]; Denisov and Afanas'ev, 2005[15]; Oz et al., 2005[44]). The excessive production of oxidizing agents in the absence of an effective defensive system may, in the long term, trigger a cascade of oxidative reactions highly harmful to cells, there by compromising their integrity. However, not all free radicals have to be considered harmful to the body; in fact, a small amount is normally produced and plays a protective role for the organism. Numerous biochemical processes in our organism continuously produce ROS and other RS (Uttara et al., 2009[61]), and certain amounts of oxidizing substances are essential to maintain the correct cell functioning and the mechanisms of homeostasis (Alkadi, 2020[4]).

### Oxidant/antioxidant balance

In physiological conditions there is an antioxidant barrier characterized by a balance between the quantity of free radicals that are produced and the quantity of free radicals that are physiologically eliminated from the body. The antioxidant barrier plays a key role against free radicals; the human body has highly complex antioxidant defence systems that cooperate with other cellular protection systems against the oxidative damage. The enzymatic system with the highest antioxidant activitis represented by the superoxide dismutase (SOD), belonging to a family of metallo-enzymes whose function is to eliminate the radical anion superoxide. Another antioxidant system is represented by the glutathione (GSH) peroxidase (GPx), which has a reductive action against organic hydroperoxides using GSH as co-substrate. Furthermore, catalase (CAT) induces a reduction of hydrogen peroxide (Holley et al., 2011[25]; Li, 1995[33]). 

SOD is an important physiological antioxidant which is found in three isoforms: extracellular superoxide dismutase (ECSOD), manganese superoxide dismutase (MnSOD), and copper-zinc superoxide dismutase (Cu/ZnSOD). MnSOD is mainly present in mitochondria, while Cu/ZnSOD is found in the cytosol. In situations of oxidative stress, when an increase in H_2_O_2 _is recorded, Cu/ZnSOD enters the cell nucleus where it carries out its antioxidant activity through gene regulation (Holley et al., 2011[25]; Li, 1995[33]; Vanfleteren, 1993[63]; Reveillaud et al., 1991[47]; Tsang et al., 2014[58]). The main source of redox species is the mitochondrial respiratory chain while MnSOD is the major mitochondrial antioxidant enzyme. Cu/ZnSOD has two identical sub-units of approximately 32 kDa, each containing a metal cluster. The active site consists of a copper atom and a zinc atom (Vanfleteren, 1993[63]; Reveillaud et al., 1991[47]; Tsang et al., 2014[58]).

In addition, the non-enzymatic antioxidants are divided into directly or indirectly acting antioxidants. The former include lipoic and carotenoids, and play a relevant role in the protection against oxidative stress. The latter comprise chelating and binding agents, which can reduce metal ions and prevent radical formation (Uttara et al., 2009[61]). An important group of non-enzymatic antioxidants which play a protective role against the damaging effects of ROS are the thiol group (-SH) containing molecules (Valko et al.,2006[62]). The α-lipoic acid (ALA) is a disulfide derived from octanoic acid that is considered a free radical scavenger and a very important antioxidant thiol. Indeed, ALA possesses two oxidized or reduced thiol groups capable of neutralizing diverse reactive species of ROS and RNS. Therefore, ALA can rapidly induce an increase of the intracellular GSH and ascorbate levels in the liver, which usually tend to decrease with age (Nebbioso et al., 2013[41]; Lykkesfeldt and Ames, 1999[38]).

## Oxidative Stress and the Visual System

Different types of tissues respond differently to the action of oxidative stress. In particular, the tissues of the central nervous system are highly sensitive to oxidative damage because they are characterized by a low level of antioxidant enzymes and a high content of oxidizable substrates. Furthermore, neuro-chemical reactions produce numerous free radicals. The oxidative stress has the undesirable effect of breaking a biochemical balance, can affect the onset or course of a large number of pathologies, and is responsible for both premature aging and neurodegeneration (Sies, 1993[49]; Tangvarasittichai and Tangvarasittichai, 2018[53]). Vitamins, polyphenols, and trace elements also intervene in this important defensive activity. In specific conditions, free radicals can cause cell and tissue damage, inducing chronic inflammatory reactions such as the typical alterations affecting the eye and the entire visual system (Ung et al., 2017[59]). Indeed, given its anatomical structure and constant contact with the external environment the eye is undoubtedly one of the organs most widely affected by free radicals (Tangvarasittichai and Tangvarasittichai, 2018[53]; Ung et al., 2017[59]; Nebbioso et al., 2013[42], 2012[40]). The appearance of inflammatory processes affecting the eye and the exposure to various types of radiations or chemical agents can be directly responsible for the high increase in hydrogen peroxide in the eye, with a consequent increase in ROS responsible for oxidative action. The oxidative action exerted by free radicals at ocular level manifests as structural and functional alterations in the corneal epithelium associated with apoptotic processes in which keratocytes and stromal fibroblasts play a central role (Ung et al., 2017[59]; Taurone et al., 2020[54]). In the light of such scientific evidence, the aim of this review was to evaluate the antioxidant effects of ALA and SOD in the visual system, and the possible induction capacity of tissue regeneration following neurodegenerative damage caused by the accumulation of free radicals.

### Mechanisms of oxidative damage to the visual system

Oxidative stress is believed to be one of the mechanisms responsible for the cellular and neuronal damage observed both during the normal aging process and during development of neurodegenerative diseases (Good el al., 1996[19]) and affects all tissues including those of the visual system. Consequently, the role of oxidative stress continues to be the subject of numerous studies. It has long been established that an increase in reactive chemical species leads to damage at the level of biological macromolecules, including nucleic acids (DNA and RNA), lipids, and proteins. Once this damage has been triggered, its role in aging and neurodegenerative disorders may be critical. Indeed, many authors have suggested a correlation between increased damage, defects in the DNA repair system, aging, and neurodegenerative diseases (Du et al., 2009[17]). It has been demonstrated that DNA oxidation increases with age, with higher levels in the cerebral cortex and cerebellum of elderly subjects (Butterfield et al., 2007[8]). 

An imbalance of the homeostatic mechanisms that regulate the equilibrium between oxidizing and antioxidant substances results in oxidative damage (Alderton et al., 2001[3]), and this imbalance also plays a fundamental role in the etiology of a great variety of inflammatory and neurodegenerative disorders (Abushouk et al., 2017[2]; Halliwell and Gutteridge, 2015.[22] Nowadays, growing attention is being paid to the cellular aging process and to the underlying factors (Hase et al., 2018[24]; Davalli et al., 2016[13]; Stefanatos and Sanz, 2018[51]), in an attempt to clarify the involutional and degenerative pathologies occurring in old age, which affect various organs and systems. In this context, the neurodegenerative processes impacting the visual system assume particular importance, owing to the frequency and the severity of the disability they may cause (Taurone et al., 2020[54]; Babizhayev and Yegorov, 2016[5]). The expression of the alterations and the severity of damage ranges from slight modifications of the main metabolic functions to more severe modifications of neurotransmitters with impairment of the neuronal function. The exact molecular mechanisms underlying these processes have still not been entirely clarified and little is known regarding their temporal sequence. In the light of these uncertainties, the etiopathogenesis of neurodegenerative processes must be considered heterogeneous and multifactorial. Recent experimental studies have been performed to understand whether the neurodegeneration mechanisms identified are specific for a single pathology or common to different conditions. Recently, the role played by oxidative stress in the pathogenesis of neurological disorders has been the subject of great interest. There is strong evidence that free radicals are involved in the onset and progression of many eye diseases (Ung et al., 2017[59]). Inflammation and oxidative stress have a crucial role in the etiology and progression of age-related eye diseases, which are the leading causes of blindness and include glaucoma, age-related macular degeneration, diabetic retinopathy, and dry eye disease (Taurone et al., 2015[56], 2019[57], 2020[55]; Fehér et al., 2018[18]). In recent years, many studies have focused their attention on phytochemical compounds with anti-inflammatory and antioxidant properties, which can be advantageously employed for treating and, in particular, for preventing several eye diseases. Some phytochemical compounds, such as carotenoids, resveratrol, and curcumin, can lower the production of ROS and consequently inhibit the activity of VEGF and TNF-α, as well as of other inflammatory cytokines (Rauf et al., 2017[45]; London and Beezhold, 2015[35]; Abu-Amero et al., 2016[1]). The term “antioxidant” covers all those molecules capable of stabilizing or deactivating free radicals before they produce cell damage. Several experimental models have been introduced to better understand the mechanisms responsible for the damage produced by ischemic phenomena affecting the retina. Numerous *in vitro* models of damage to retinal ganglion cells (RGC) based on the reduction of oxygen and glucose availability have been described in literature (Izumi et al., 2003[26]; Kinukawa et al., 2005[27]; Mastrodimou et al., 2005[39]). Ischemic diseases of the central nervous system are mediated by a complex cascade of biochemical events in which the excitatory neurotransmitter glutamate is a common element and plays a central role in the degenerative process. This is also true for the retina indeed, the excessive activation of NMDA (*N*-methyl-*D*-aspartate) and non-NMDA receptors, as well as the consequent accumulation of nitric oxide, are among the mechanisms responsible for the cell loss induced by ischemic phenomena. Furthermore, this loss may be prevented by systemic pre-treatment with NMDA and non-NMDA receptor antagonists and with *L*-NAME (*L*-Nitroarginine methyl ester) (Nucci et al., 2007[43]). In metabolic terms, the eye is a very active structure. Since it is mostly exposed to light, oxidative and, particularly, photo-oxidative processes play a critical role in the pathological conditions of the eye, in particular the ones associated with aging (Williams, 2008[64]).

A variety of interconnected pathways may lead to generation of ROS in the eye. The normal process of electron transport and the activity of cytochrome P450 can generate the superoxide anion. The production of free radicals may be inhibited by the enzymatic activity of molecules such as SOD, CAT, and GPx by restoring the body homeostasis (Lightfoot et al., 2006[34]). Oxidative stress is the result of free radicals failing to be inhibited by antioxidant enzymes (Zanza et al., 2019[68]). This deficit induces cellular oxidation which leads to tissue necrosis (Remacle et al., 1995[46]). This process becomes particularly accentuated with aging, where the damage induced by oxidative stress increases and the normal cell regeneration processes are less active (Cabrera and Chihuailaf, 2011[9]; Carneiro and Andrade, 2017[12]).

**Figure 1:**
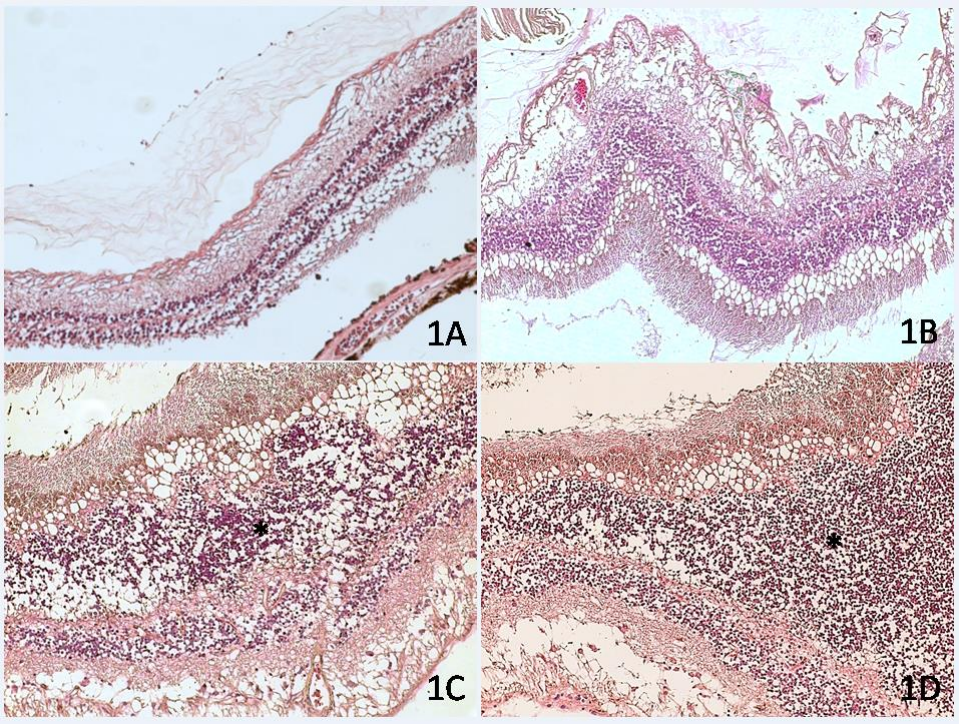
Human retina of patients affected by diabetic retinopathy. H&E stain. Within the retinal tissue, some microhemorrhages and cystoid degeneration of the inner layers of the retina with cellular apoptosis (C and D, asterisk) are visible. (1A) magnification 10×. (1B) Magnification 20 x; (1C-1D) Magnification 40 x.

Oxidative stress plays an important role also in the development of diabetic retinopathy (Kubes, 2000[29]). The increase in blood glucose concentration determines numerous metabolic imbalances that contribute to the production of free radicals responsible for oxidative stress (Lopez-Galvez et al., 2014[37]). The high exposure of the delicate retinal tissues to the action of free radicals induces an increase in inflammatory cytokines such as IL-1β and TNF-α which act on the vascular cells inducing angiogenesis at the level of retinal lesions (Li et al., 2017[31]). The retinal photoreceptors carry out an intense metabolic activity and are therefore continuously exposed to the damage induced by free radicals (Figure 1(Fig. 1)). Following ischemia, a high amount of ROS is produced within the retina, with a consequent increase of damage induced by oxidative stress and loss of retinal cones (Gopinath et al., 2018[20]; Campochiaro and Mir, 2018[10]).

### The role of antioxidant molecules in the visual system

The aim of our study was to identify the factors responsible for progression of age-related inflammatory eye diseases in order to prevent the irreversible damage that causes blindness. Considering this, we have focused our attention on the antioxidant activity of ALA and SOD in the visual system. The aged tissues, namely those prone to neurodegeneration, significantly express iNOS. Once produced, NO can cause irreversible tissue injuries (Campochiaro et al., 2015[11]; Wu and Rao, 2008[67]; Steinert et al., 2010[52]). The toxic effects of iNOS have been documented in a wide range of degenerative and inflammatory disorders, including Parkinson's disease, Alzheimer's disease, and multiple sclerosis (Wu and Rao, 2008[67]; Upadhyay and Dixit, 2015[60]). The antioxidant action of SOD and GST enzymes seems to inhibit the production of ROS; moreover, the enzymatic activity of these enzymes has a protective action on the neurons (Simon et al., 2000[50]). Studies regarding iNOS over-expression in mice have demonstrated that increased NO levels could cause retinal photoreceptor apoptosis death (Wright et al., 2010[65]). ROS may induce or prevent the apoptotic process and such activity depends on their intracellular concentration (Handa, 2012[23]). Treatment with antioxidant molecules leads to a reduction in the oxidative damage (Wright et al., 2010[65]; Handa, 2012[23]). Furthermore, an increase in the stability of mitochondrial membranes, as shown by the diminution of the LOP reaction induced by free radicals, could be useful to reduce the release of some pro-apoptotic molecules that are responsible for the induction of the programmed cell death.

Some reports indicate that oxidative stress induces a deficiency of MnSOD and consequently an increase in the apoptotic process (Kokoszka et al., 2001[28]). Considering that ALA is also able to reduce cellular apoptosis, the combined use of the two agents seems to greatly strengthen their antioxidant effects, most likely as a consequence of a synergistic action of ALA and SOD. In particular, considering that SOD has an extracellular activity and ALA mainly an intracellular one (Nebbioso et al., 2013[42], 2012[40]; Bertolotto and Massone, 2012[6]), their combined use for anti-apoptotic purposes could be useful in reducing the neurodegeneration at retinal level.

## Conclusions

Oxidative stress has a notable role in pathological conditions affecting the visual system. Light passes through all layers of the eye and generates high amounts of ROS during aging. If this condition is accompanied by pathological stimuli such as high IOP or increase blood sugar concentrations, there is an even greater production of ROS. Some studies have reported that the onset of eye diseases is more frequent when there is an imbalance between the production of free radicals and antioxidants by the body's defence mechanisms (Di Marco et al., 2015[16]).

Another step of damage could be the imbalance of cerebral autoregulation, a mechanism that prevents the accumulation of free radicals overall in the central nervous system where the blood stream is finely regulated (Longhitano et al., 2021[36]).

From the data available in the literature, it appears evident that during physiological aging the neuronal cells of the eye undergo a neurodegenerative process mainly due to the accumulation of free radicals, especially considering that the normal antioxidant defence systems become less effective with age. In this context, the use of antioxidant molecules such as ALA and SOD could significantly protect against retinal degeneration. Consequently, there would be an improvement in the conditions of the ocular nervous tissue. Further future studies are needed not only for a broader case analysis but also to evaluate other markers capable of inducing oxidative stress to define whether oxidative damage is an immediate cause or a consequence in the pathogenesis of ocular diseases.

## Notes

Samanta Taurone, Massimo Ralli, Marco Artico, Marcella Nebbioso, Roberta Costi and Alessandra Micera contributed equally to this publication.

## Declaration

### Funding

This paper was financially supported by Ministry of Health (grant no. RC 2765943) and Fondazione Roma*.*

### Author contributions

Conceptualization, S.T., M.R. and M.A.; methodology, V.M.N. software, S.S.; validation, A.M., M.B. and P.F., investigation, M.N., data curation, A.M. and S.A.N.; writing-original draft preparation, S.T., M.R. and. A.M., writing-review and editing, M.A. and S.A.N.; visualization, R.C.; supervision, A.M.; funding acquisition, A.M. All authors have read and agreed to the published version of the manuscript.

### Institutional review board statement

Not applicable. 

### Informed consent statement

Not applicable.

### Data availability statement

Not applicable.

### Conflict of interest

The authors declare no conflict of interest.
